# Association of cigarette design features with smoker characteristics and risk beliefs: Cross-sectional findings from the 2019 ITC France Survey

**DOI:** 10.18332/tpc/209142

**Published:** 2025-09-30

**Authors:** Parker A. Polston, Anne C. K. Quah, Geoffrey T. Fong, Richard J. O’Connor

**Affiliations:** 1 Department of Health Behavior, Roswell Park Comprehensive Cancer Center, Buffalo, United States; 2 Department of Psychology, University of Waterloo, Waterloo, Canada; 3 School of Public Health Sciences, University of Waterloo, Waterloo, Canada; 4 Ontario Institute of Cancer Research, Toronto, Canada

**Keywords:** cigarettes, smoking, design features, filter ventilation, intention to quit, risk beliefs

## Abstract

**INTRODUCTION:**

Despite the lack of evidence supporting an association between certain cigarette design features (e.g. filter ventilation) and harm reduction, such features often perpetuate false perceptions of safety among people who smoke. Evaluating how product characteristics shape perceptions and behaviors can help clarify these misconceptions and support the importance of restricting deceptive manufacturing. We explore relationships between cigarette design features and perceptions of smoothness and harm, as well as intention to quit.

**METHODS:**

Cigarette brand/variety and consumer perceptions/behaviors data come from the 2019 ITC France Survey, which was administered to a nationally representative sample of French adults. This cross-sectional secondary analysis incorporated cigarette product information reported to the Agency for Food, Environmental and Occupational Health & Safety in 2021. Logistic regression analyses were done using SPSS V27.

**RESULTS:**

Greater own brand cigarette filter length (mm) (adjusted odds ratio, AOR=1.11; 95% CI: 1.05–1.17) was significantly associated with higher odds of perceiving one’s own brand as smoother than other brands, while greater open pressure drop (mmWG) (AOR=1.02; 95% CI: 1.00–1.05) was associated with perceiving own-brand as safer than other brands. Respondents who described themselves as being in poor or fair health (vs good health) were more likely to perceive smooth/ultra (AOR=1.70; 95% CI: 1.22–2.37) and their own cigarettes (AOR=1.76; 95% CI: 1.05–2.95) as less harmful, as well as less likely to perceive their own brand as smoother (AOR=0.66; 95% CI: 0.47–0.93). Male (vs female) respondents were more likely to perceive smooth/ultra (AOR=1.88; 95% CI: 1.38–2.55) and their own cigarettes (AOR=1.89; 95% CI: 1.12–3.19) as less harmful.

**CONCLUSIONS:**

We found evidence that certain design features and participant characteristics are associated with misconceptions regarding the smoothness and safety of cigarettes. These findings support greater monitoring of potentially deceptive product characteristics.

## INTRODUCTION

Despite substantial progress in reducing prevalence, cigarettes remain the most used tobacco product and carry the greatest association with morbidity and mortality^1^. Cigarette manufacturers continue to introduce new brand varieties to retain people who currently smoke and attract new users. Specific design features such as filter ventilation contribute to sensory experiences during smoking and may convey perceptions of reduced health risk^2^. Filter ventilated cigarettes, however, do not promote risk reduction, as their use elicits compensatory behavior, including taking larger puffs and blocking vents^2^. Additionally, in the US, findings from the 2007–2012 NHANES cycles demonstrate that cotinine levels (a biomarker for nicotine exposure) do not differ by brand, tar group, or menthol status, suggesting that design features may have a limited capacity in reducing toxicant exposure^3^. In compliance with Framework Convention on Tobacco Control, countries have removed many outward indicators of distinctions among brands through standardized packaging and large pictorial health warnings^4^. France is no exception, having implemented these in 2017^5^. As an EU Member State, manufacturers in France must also comply with the 10-1-10 standard, which was required under the Tobacco Products Directive (2014/40/EU), which came into force in 2014, capping tar, nicotine, and CO emissions^6^. Filter ventilation is a key means of achieving compliance. Despite the lack of evidence supporting harm reduction, and the subsequent banning of misleading descriptors, manufacturers continue to market products in a way that may perpetuate misconceptions^7-13^.

Cigarette packaging, in its display of certain color-schemes, has been found to be associated with specific product design characteristics. In fact, less saturated and brighter packaging has demonstrated consistency with greater filter ventilation^14^. In recognition of these seemingly deceptive marketing tactics, understanding how filter ventilation and other design features shape consumer perceptions and experiences is important. A series of studies, primarily but not exclusively in the US, have explored the relationship between specific cigarette design features, including filter ventilation, and consumer behaviors and perceptions^15-24^. However, little work has been done with respect to cigarette sensory perceptions in a plain-packaging context and an emissions cap.

Several studies have demonstrated evidence that highly ventilated cigarettes are linked to perceptions of lower health risk^18-22^, though not all are in support of this finding^23^. Furthermore, cigarette brands believed to be ‘light’ – a descriptor commonly used in correspondence with ventilation – have been perceived by consumers as less harmful than regular cigarettes^16,17^. Filter ventilation also contributes to sensory experiences, such that cigarettes with greater ventilation are largely perceived as less intense^23^, smoother^15,20,23^, and lighter^2,15^. Additionally, in a US sample of young adults, cork-tipped ventilated cigarettes were situated as the best tasting and most attractive when compared to unfiltered and white-tipped ventilated cigarettes, suggesting that sensory experiences might also vary between types of vented cigarettes^21^. Some studies, however, have published null or dissimilar findings, with ventilation not being associated with perceptions of lightness^24^ or smoothness^15^, and being associated with less taste enjoyment^23^.

In recognition of these discrepant findings, further evaluation of the impact of product design features on characteristics and risk beliefs of people who smoke is warranted. In this analysis, we aim to explore the relationship between several design features and perceptions of smoothness and harm, as well as intention to quit smoking. We hypothesized that those using products with higher filter ventilation would perceive them as less harmful and smoother.

## METHODS

### ITC France survey

This study is a cross-sectional, secondary data analysis of the ITC France Survey^25,26^, which was administered to a nationally representative sample of French adults (≥18 years) who do (defined as smoking at least monthly) and do not smoke (defined as not smoking at all). Fieldwork was conducted from 31 October to 17 December 2019, by Rakuten Insight using a web-based questionnaire. The sampling frame of panelists was nationally representative of French adults who did and did not smoke, with quotas based on region of residence, sex, and age based on French census data. Exclusion criteria were being aged <18 years, reporting less than monthly tobacco use, and meeting definition of a ‘speeder’ (completing the survey too quickly). Additional details about the survey sampling and data cleaning can be found in the Technical Report^26^. The current analysis included only respondents who both smoked and were asked questions regarding their knowledge, attitudes, beliefs, perceptions, behaviors, and use patterns of cigarettes. To determine usual cigarette brand, respondents were asked: ‘Do you have a usual brand and variety of cigarettes? We mean the brand that you smoke more than any other. Yes/No/Don’t know/Refused]’. Those who responded yes were subsequently asked: 1) ‘Is your usual brand of cigarettes…? [Factory-made cigarettes/Rolling tobacco/Refused/Don’t know]’; 2) ‘What is your usual brand of factory-made cigarettes? List of coded brand responses/Other brand (not listed)/Refused/Don’t know]’; and c) ‘What is your usual brand variety of those factory-made cigarettes? [List of coded brand variety responses/Other brand variety (not listed)/Refused/Don’t know]’.

### Outcome measures

We were interested in four primary outcomes related to cigarette product perceptions and behaviors. Relative harm was assessed by the item: ‘Do you think that the brand you usually smoke might be a little less harmful, no different, or a little more harmful, compared to other cigarette brands? [A little less harmful/No different/A little more harmful/Refused/Don’t know]’. Relative smoothness was assessed by: ‘Thinking about the cigarettes you usually smoke compared to other cigarettes, are your cigarettes harsher or smoother on your throat? [Harsher/About the same/Smoother /Refused/Don’t know]’. The perceived relationship between smoothness and harm was assessed by the item: ‘Do the words ‘smooth’ or ‘ultra’ in the name help to indicate whether a cigarette or roll-your-own brand could be less harmful compared to others? [Not at all/A little/Somewhat/A lot/Not applicable/Refused/Don’t know]’. Finally, participants reported whether they planned to quit smoking within the next month, 1–6 months from now, sometime in the future, or not planning to quit.

### Covariate measures

Participants reported sex (male, female) and age was categorized into four groups (18–34; 35–44; 45–45; ≥55 years) for analysis. Cigarettes smoked per day were assessed as a number and categorized (<10; 11–20; ≥21) for analysis. Education level was categorized as low (less than high school), moderate (high school graduate/equivalent or some university), or high (university graduate or advanced degree) consistent with ITC procedures. Self-rated health was assessed as excellent, very good, good, fair, or poor, dichotomized as fair or poor versus good or better for analysis.

### Cigarette design

Under the European Union Tobacco Products Directive (2014/40/EU), manufacturers are required to disclose specific product information to member states, via the EU Common Entry Gate^27^. Data for the French market were obtained from the Agency for Food, Environmental and Occupational Health & Safety (ANSES) for 2021^28^. This dataset included product name and subtype, identifier number (e.g. SKU), diameter (mm), total weight (mg), tobacco weight (mg), filter length (mm), filter ventilation (%), open and closed pressure drop (mmWg), and ISO tar, nicotine, and CO emissions. A total of 3138 brand styles were included in the dataset.

Product characteristics data were matched to ITC self-reported brand and variety. Of 2212 respondents in the survey, 1679 were adults (aged ≥18 years) who currently smoked cigarettes. Of these, 214 (12.7%) who used roll-your-own cigarettes exclusively were excluded. Of the remaining respondents, 1287 (88%) reported having a usual factory-made brand. Of these, 1039 (81%) reported using a cigarette brand that could be matched to a brand style from the ANSES list. Key reasons for lack of match were reporting a brand not on the ANSES list, or insufficient detail to match to a particular brand style (Supplementary file Table S1).

### Data analysis

The analytic sample was characterized using descriptive statistics. Bivariate relationships among respondent demographic factors (age, gender, education level, cigarettes per day, perceived health status), cigarette characteristics, and outcome measures were assessed using cross-tabulations with chi-squared, ANOVA, or correlations. Variables for which bivariate relationships were significant were included in subsequent stepwise logistic regression models examining the effects of cigarette characteristics measures on outcomes, adjusting for demographic factors. Adjusted odds ratios (AORs) and their 95% confidence intervals (CIs) are reported, adjusted for all covariates included in the respective models. Additionally, a mediation analysis was performed using the Baron and Kenny approach to evaluate whether perceiving one’s own brand as smoother mediates the relationship between product design feature and perceiving one’s own brand as less harmful^29^. Participants with missing values were excluded. All analyses were weighted and performed using SPSS V27, with p<0.05 indicating statistical significance.

## RESULTS

Table 1 presents the sociodemographic and behavioral profile of the survey respondents for whom brand characteristics data were available. Most of the sample was aged ≤44 years, male, of low to moderate education level, smoked ≤10 cigarettes per day, and perceived themselves as in at least good health. Marlboro was the most commonly reported brand family at nearly a quarter of respondents. The typical cigarette used by respondents had on average 10 mg tar, 0.7 mg nicotine, 28% filter ventilation, was 83 mm long, and contained 815.5 mg tobacco filler.

**Table 1:** Participant characteristics and cigarette design features, 2019 ITC France Survey (N=1218)

*Characteristics*	*n*	%*
**Age** (years)		
18–34	480	39.4
35–44	254	20.8
45–54	252	20.7
≥55	232	19.0
Missing=0		
**Gender**		
Male	630	51.7
Female	588	48.3
Missing=0		
**Education level**		
Low (no education, some high school)	578	48.4
Moderate (completed high school, some university)	443	37.1
High (completed university, PhD and/or postdoctoral)	174	14.5
Missing=23		
**Cigarettes per day**		
0–10	665	55.5
11–20	442	36.9
≥21	90	7.5
Missing=20		
**Perceived health status**		
Poor or fair	370	31.4
Other (good, very good, or excellent)	807	68.6
Missing=41		
**Usual brand of factory-made cigarettes**		
Marlboro	286	23.6
Winston	143	11.8
Lucky Strike	129	10.6
Philip Morris	116	9.5
Camel	109	9.0
Other	429	35.5
Missing=3		
***Cigarette design features***	***Mean***	***SD***
**Tar** (mg)	9.1	1.6
Missing=244		
**Nicotine** (mg)	0.7	0.1
Missing=244		
**CO** (mg)	9.1	1.5
Missing=244		
**Pressure drop open** (mmWG)	85.4	16.0
Missing=244		
**Pressure drop closed** (mmWG)	94.0	37.3
Missing=244		
**Ventilation** (0–100%)	30.2	13.3
Missing=244		
**Product length** (mm)	84.3	6.6
Missing=244		
**Diameter** (mm)	7.7	0.3
Missing=244		
**Wet tobacco weight** (mg)	841.2	78.0
Missing=244		
**Dry tobacco weight** (mg)^a^	622.8	67.1
Missing/excluded=256		
**Filter length** (mm)^a^	23.6	3.0
Missing/excluded=308		

a Data beyond three standard deviations of the mean were excluded.

* Valid percentage.

We explored relationships among the cigarette design features. As anticipated, filter ventilation was strongly negatively related to tar (r= -0.42, p<0.001), while dry weight was positively associated with tar (r=0.29, p<0.001). Interrelationships among other measures are shown in Supplementary file Table S2.

Supplementary file Tables S3A and S3B show mean values for cigarette design features by participant demographics and outcome variables, along with associated ANOVA tests. Pressure drop, ventilation, weight, and length showed consistent relationship to multiple person-level factors. Compared with older age groups, individuals aged 18–34 years reported utilizing cigarettes that on average had lower open pressure drop (82.97 mmWG, p=0.002), closed pressure drop (87.32 mmWG, p<0.001), ventilation (28.0%, p<0.001), overall length (83.07 mm, p<0.001), and wet tobacco weight (832.07 mg, p=0.001). Filter length (23.25 mm, p<0.001) and dry tobacco weight (614.57 mg, p=0.05) were lowest in cigarettes reported by those aged 35–44 years, and diameter (7.68 mm, p=0.02) was lowest in cigarettes reported by those aged ≥55 years. Cigarettes reported by male respondents tended on average to have lower closed pressure drop (87.6 mmWG vs 100.7 mmWG, p<0.001), ventilation (28.5% vs 31.9%, p<0.001), filter length (23.3 mm vs 23.9 mm, p=0.01) and overall length (83.7 mm vs 84.9 mm, p=0.01), as well as larger diameter (7.8 mm vs 7.7 mm, p=0.001). Individuals who report smoking ≤10 cigarettes per day, utilize cigarettes that, on average, have a lower diameter (7.71 mm, p=0.01), wet tobacco weight (833.12 mg, p=0.001), and dry tobacco weight (617.01 mg, p=0.01) when compared with those who smoke more, 11–20 and 21 cigarettes per day. Cigarettes reported by individuals with a low level of education tended to have lower open pressure drop (84.28 mmWG, p=0.03) compared to those reported by individuals with moderate or high level education.

With respect to outcome measures, perceived own-brand harshness was significantly associated with closed pressure drop, ventilation, overall length, and filter length. Perceived harmfulness of own brand was associated with open pressure drop, diameter, wet weight, and dry weight. Perception that smooth/ultra cigarettes are less harmful was associated with wet weight. Intention to quit was not associated with any cigarette design features.

Supplementary file Table S3C displays mean values for tar, nicotine, and CO (ppm), by participant demographics and outcome variables, along with associated ANOVA tests. Interestingly, cigarettes reported by those who perceive themselves as being in poor or fair health tended to emit more CO than cigarettes reported by individuals who perceive themselves as being in at least good health (9.24 vs 9.01, p=0.02).

Table 2 presents results of a stepwise logistic regression model of factors associated with the perception that own brand was smoother than other brands. Initial bivariate analyses indicated that closed pressure drop, ventilation, overall length, and filter length were associated with this outcome. However, stepwise results indicate that only filter length should be retained in the model, and that each 1 mm increase in filter length is associated with 1.11 times greater odds of perceiving own brand as smoother than other brands (p<0.001). Those who reported poor or fair health were significantly less likely to report that their own brand was smoother (AOR=0.66, p=0.02). Age, gender, education level, and cigarettes per day were not significantly related to the perception of own brand as smoother.

**Table 2:** Stepwise logistic regression of factors associated with perceiving own brand of cigarette as smoother than other brands, 2019 ITC France Survey (N=821)

*Variables*	*AOR (95% CI)*	*Wald χ^2^*	*p*
**Filter length** (mm)	1.11 (1.05–1.17)	15.70	<0.001
**Age** (years)			
18–34	1.08 (0.69–1.67 )	0.11	0.75
35–44	0.85 (0.52–1.38 )	0.43	0.51
45–54	0.71 (0.43–1.16 )	1.84	0.18
≥55 ®	1		
**Gender**			
Male	0.89 (0.65–1.21 )	0.54	0.46
Female ®	1		
**Education level**			
Low (no education, some high school)	1.16 (0.73–1.83 )	0.40	0.53
Moderate (completed high school, some university)	1.01 (0.64–1.61 )	0.00	0.95
High (completed university, PhD and/or postdoctoral) ®	1		
**Cigarettes per day**			
0–10 ®	1		
11–20	1.14 (0.82–1.58 )	0.63	0.43
≥21	0.94 (0.49–1.81 )	0.04	0.85
**Perceived health status**			
Poor or fair	0.66 (0.47–0.93 )	5.76	0.02
Other (good, very good, or excellent) ®	1		

AOR: adjusted odds ratio. Model adjusted for age, gender, education level, cigarettes per day, perceived health status, and filter length. ® Reference categories.

Table 3 presents results of a logistic regression model of factors associated with the perception that smooth/ultra cigarettes are less harmful. Initial bivariate analyses indicated that wet tobacco weight was associated with this outcome, but logistic modeling suggests that it was not significantly associated (p=0.16), after controlling for other factors. Younger respondents (18–34 years, AOR=2.69; 35–44 years, AOR=2.37) were significantly more likely than those aged ≥55 years to believe smooth/ultra cigarettes were less harmful. Those who reported poor or fair health (AOR=1.70, p=0.002) and males (AOR=1.88, p<0.001) were also significantly more likely to believe that smooth/ultra cigarettes were less harmful.

**Table 3:** Logistic regression of factors associated with perceiving smooth/ultra cigarettes as less harmful, 2019 ITC France Survey (N=749)

*Variables*	*AOR (95% CI)*	*Wald χ^2^*	*p*
**Wet tobacco weight** (mg)	1.00 (1.00–1.00)	1.98	0.16
**Age** (years)			
18–34	2.69 (1.71–4.25)	18.15	<0.001
35–44	2.37 (1.43–3.92)	11.29	0.001
45–54	1.13 (0.67–1.89)	0.21	0.65
≥55 ®	1		
**Gender**			
Male	1.88 (1.38–2.55)	16.21	<0.001
Female ®	1		
**Education level**			
Low (no education, some high school)	1.41 (0.90–2.19)	2.27	0.13
Moderate (completed high school, some university)	1.30 (0.83–2.04)	1.30	0.25
High (completed university, PhD and/or postdoctoral) ®	1		
**Cigarettes per day**			
0–10 ®	1		
11–20	0.76 (0.55–1.05)	2.79	0.09
≥21	0.74 (0.41–1.34)	0.98	0.32
**Perceived health status**			
Poor or fair	1.70 (1.22–2.37)	9.83	0.002
Other (good, very good, or excellent) ®	1		

AOR: adjusted odds ratio. Model adjusted for age, gender, education level, cigarettes per day, perceived health status, and wet tobacco weight. ® Reference categories.

Table 4 presents results of a stepwise logistic regression model of factors associated with the perception that own brand was less harmful than others. Initial bivariate analyses indicated that open pressure drop, diameter, wet weight, and dry weight were associated with this outcome, but logistic modeling suggests that only open pressure drop was significantly associated (p=0.02), after controlling for other factors. Specifically, for every 1 mmWG increase in pressure drop, odds of believing own brand was safer increased by 1.02 times. Those aged 45–54 years were significantly less likely than those aged 55 years to believe their brand cigarettes were less harmful. Male respondents were nearly twice as likely to believe their own brand was less harmful (AOR=1.89, p=0.02). Finally, those who reported poor or fair health were also significantly more likely to believe their own brand was less harmful (AOR=1.76, p=0.03). Supplementary file Table S4 reports findings for intention to quit; ventilation was not independently associated with quit intentions.

**Table 4:** Stepwise logistic regression of factors associated with perceiving own brand of cigarette as less harmful than other brands, 2019 ITC France Survey (N=832)

*Variables*	*AOR (95% CI)*	*Wald χ^2^*	*p*
**Pressure drop open** (mmWG)	1.02 (1.00–1.05)	5.19	0.02
**Diameter** (mm)	0.63 (0.32–1.21)	1.96	0.16
**Age** (years)			
18–34	0.63 (0.33–1.20)	2.01	0.16
35–44	0.87 (0.45–1.72)	0.15	0.70
45–54	0.25 (0.10–0.65)	8.13	0.004
≥55 ®	1		
**Gender**			
Male	1.89 (1.12–3.19)	5.71	0.02
Female ®	1		
**Education level**			
Low (no education, some high school)	0.93 (0.47–1.87)	0.04	0.84
Moderate (completed high school, some university)	0.68 (0.33–1.43)	1.03	0.31
High (completed university, PhD and/or postdoctoral) ®	1		
**Cigarettes per day**			
0–10 ®	1		
11–20	0.90 (0.54–1.50)	0.16	0.68
≥21	0.08 (0.00–1.46)	2.92	0.09
**Perceived health status**			
Poor or fair	1.76 (1.05–2.95)	4.57	0.03
Other (good, very good, or excellent) ®	1		

AOR: adjusted odds ratio. Model adjusted for age, gender, education level, cigarettes per day, perceived health status, pressure drop open, and diameter. ® Reference categories.

Table 5 presents the results of a mediation analysis exploring whether perception that own brand was smoother mediates relationships between product design features and perception that own brand was less harmful, while adjusting for demographic factors. Perceiving one’s own brand as smoother fully mediated this relationship for several product design features, including ventilation, closed pressure drop, product length, and filter length. Mediation was strongest with filter length as the predictor, as demonstrated by the indirect effect of 0.13. Additionally, perceiving one’s own brand as smoother was found to partially mediate the relationship between open pressure drop and perceiving one’s own brand as less harmful.

**Table 5:** Mediation analysis of perceiving own brand of cigarette as smoother than other brands, 2019 ITC France Survey

*Cigarette design features*	*Model 1^a^*			*Model 2^b^*			*Indirect effect*
	*n*	*β*	*p*	*n*	*β*	*p*	
Ventilation (0–100%)	821	0.02	0.002	770	1.23	<0.001	0.02^c^
Pressure drop open (mmWG)	822	0.01	0.027	770	1.23	<0.001	0.01^d^
Pressure drop closed (mmWG)	822	0.01	0.016	770	1.24	<0.001	0.01^c^
Product length (mm)	822	0.04	0.002	770	1.22	<0.001	0.05^c^
Diameter (mm)	822	-0.08	0.698	771	1.29	<0.001	-0.11
Wet tobacco weight (mg)	823	0.00	0.068	770	1.31	<0.001	0.00
Dry tobacco weight (mg)	811	0.00	0.836	761	1.25	<0.001	0.00
Filter length (mm)	766	0.11	<0.001	723	1.22	<0.001	0.13^c^

a Cigarette design feature perceptions of smoother.

b Perceptions of smoother perceptions of lower harm.

c Full mediation.

d Partial mediation.

Models adjusted for age, gender, education level, cigarettes per day, and perceived health status.

## DISCUSSION

While previous studies have consistently demonstrated associations between filter ventilation and odds of perceiving one’s own brand as smoother^15,20,23^ and less harmful^18-20,22^, our results suggest that other design features consistent with the presence of ventilation may be influencing these perceptions. Adjusting for other factors, own brand cigarette filter length and open pressure drop were significantly associated with greater odds of perceiving one’s own brand as smoother and safer than other brands, respectively.

Consistent relationships between perceived health status and product beliefs are not well documented in the literature. We found health status to be significantly associated with three of our primary outcomes. Specifically, those who reported being of poor or fair health were more likely to perceive smooth/ultra and their own cigarettes as less harmful, as well as less likely to perceive their own brand as smoother. We also noted that those in poor or fair health smoked cigarettes with higher CO emissions on average. Still, it is difficult to extrapolate the direction of effect within these cross-sectional data.

Additionally, we found male respondents to be more likely to perceive smooth/ultra and their own cigarettes as less harmful. Though few studies have demonstrated significant relationships between gender and product beliefs, results from a study evaluating risk perceptions of filter ventilation in a sample of US adults suggest that females are more likely to perceive their own brand as less harmful^23^. This study also observed the odds of perceiving one’s own brand as smoother to be greater in older individuals^23^. While we did not observe a relationship between age and perceptions of own brand smoothness, we did find those aged 45–54 years to have lower odds of perceiving their own cigarettes as less harmful, and those aged 18–44 years to have greater odds of perceiving smooth/ultra cigarettes as less harmful, compared to those aged ≥55 years. Our findings are consistent with a previous study by Elton-Marshall et al.^16^, which found individuals aged ≥55 years to be more likely to perceive their own brand as less harmful.

There are several factors that might explain the observed differences between previous and current findings. First, cigarette marketing in France has been limited over the past two decades due to restrictions implemented through the Framework Convention on Tobacco Control and the European Union Tobacco Products Directive^4,6^. Additionally, due to requirements for France to comply with the EU 10-1-10 standard, design features are expected to differ from products manufactured in countries where such regulations are not in place^6^.

### Limitations

A key limitation of this study is that the matching of self-reported brands/varieties with factory-made brand names could have been imperfect (Supplementary file Table S1). Furthermore, due to the European Union Tobacco Products Directive’s limits on tar, nicotine, and CO, there is less variation in the range of product designs on the market^6^. As all respondent data are self-reported, there is potential for recall and information bias, as well as residual confounding due to unmeasured factors. Finally, although the pattern of outcomes is broadly consistent with previous studies, findings from this cross-sectional study in France may not be generalizable to other markets.

## CONCLUSIONS

Despite the banning of many deceptive descriptors, cigarettes continue to be manufactured in ways that perpetuate misconceptions. This cross-sectional study underscores the importance of monitoring certain cigarette design features that can inculcate false perceptions of smoothness and safety. Additionally, the current findings suggest that misconceptions may be more prominent in certain populations, which can be important in shaping more targeted communication efforts.

## Supplementary Material



## Data Availability

In each country participating in the international Tobacco Control Policy Evaluation (ITC) Project, the data are jointly owned by the lead researcher(s) in that country and the ITC Project at the University of Waterloo. Data from the ITC Project are available to approved researchers 2 years after the date of issuance of cleaned data sets by the ITC Data Management Centre. Researchers interested in using ITC data are required to apply for approval by submitting an International Tobacco Control Data Repository (ITCDR) request application and subsequently to sign an ITCDR Data Usage Agreement. The criteria for data usage approval and the contents of the Data Usage Agreement are described online (http://www.itcproject.org).
