# Supplementary material for: Association of cigarette design features with smoker characteristics and risk beliefs: Cross-sectional findings from the 2019 ITC France Survey
Source: Tob Prev Cessat. 2025 Sep 30;11:10.18332/tpc/209142. doi: 10.18332/tpc/209142 (PMC12489821; doi:10.18332/tpc/209142)
Supplement: Supplementary file 1 [file TPC-11-44-s1.pdf]

Supplemental Table S1. Matching self-reported brands/varieties with factory-made brand names, 2019 ITC France Survey

Factory-made Brand Names (obtained from ANSES)	Self-reported Brands/Varieties
Austin+	Austin 1839 Green, Pack Of 20<br>Austin 1839 Red, Pack Of 20<br>Austin 1839 Silver, Pack Of 20<br>Austin Blue, Pack Of 20<br>Austin Red 100, Pack Of 20
Bastos Rouge	Bastos Classic Red, Pack Of 20<br>Bastos Classic Red, Pack Of 25<br>Red Bastos, Pack Of 20
Benson & Hedges Gold	Benson & Hedges Original Gold, Pack Of 20
Benson & Hedges Gold 100’s	Benson & Hedges Original Gold 100s, Pack Of 20
Benson & Hedges Platinum	Benson & Hedges Original Platinum, Pack Of 20
Benson & Hedges Red	Benson & Hedges Original Red, Pack Of 20
Benson & Hedges Red 100's	Benson & Hedges Original Red 100s
Benson & Hedges Silver 100's	Benson & Hedges Original Silver 100s, Pack Of 20
Benson & Hedges Silver	Benson & Hedges Original Silver, Pack Of 20
Camel Filters	Camel Filters (Rigid Package), 20 Pack
Camel Essential 100's	Camel Original Essential 100s, Pack Of 20
Camel Silver	Camel Original Silver, Pack Of 20
Camel Yellow	Camel Original Yellow (Rigid Package), 20 Pack<br>Camel Yellow (Rigid Package), Pack Of 20
Camel	Original Camel, Pack Of 20
Camel Shift Fresh	Camel Shift, Pack Of 20
Chesterfield Original Red 100s 100s Hard Pack - 20 Cigarettes	Chesterfield Red 100s
Chesterfield Blue Duty Free - Hard Pack - 20 Cigarettes	Chesterfield Blue<br>Chesterfield Blue, Pack Of 20
Chesterfield Red Duty Free - Hard Pack - 20 Cigarettes	Chesterfield Red 20
Chesterfield Blue Xl Hard Pack - 25 Cigarettes	Chesterfield Blue Xl, Pack Of 25
Chesterfield Original Blue Hard Pack - 20 Cigarettes	Chesterfield Original Blue, Pack Of 20
Chesterfield Original Red Hard Pack - 20 Cigarettes	Chesterfield Original Red, Pack Of 20
Chesterfield Red Collection Hard Pack - 30 Cigarettes	Chesterfield Original Red, Pack Of 30
Chesterfield Red Xl Hard Pack - 25 Cigarettes	Chesterfield Red Xl, Pack Of 25
Chesterfield Slims Blue 100s Hard Pack - 20 Cigarettes	Chesterfield Slims Blue, Pack Of 20
Corset Ss Fp Lilas	Corsa Lilac, Pack Of 20<br>Lilac Corset, Pack Of 20
Corset Ss Fp Pink	Pink Corset, Pack Of 20
Dunhill International Red	Dunhill International Red Select, 20 Pack
Dunhill Red	Dunhill Red Select, Pack Of 20
Gauloises Blondes Bleu	Gauloises Blondes - Blue, Pack Of 25

Gauloises Blondes Bleu 100's	Gauloises Blondes Classic Blue 100s, Pack Of 20
Gauloises Blondes Bleu	Gauloises Blondes Classic Blue, Pack Of 30
Gauloises Blondes Rouge 100's	Gauloises Blondes Classic Red 100s, Pack Of 20
Gauloises Marron	Gauloises Brown Filter, Pack Of 20<br>Brown Gauloises, Pack Of 20<br>Gauloises Classic Brown, Pack Of 20<br>Gauloises Brown, Pack Of 20
Jps Firm Filter Noir 100's	Jps Classic Black 100s, Pack Of 20<br>Jps Classic Firm Filter Black 100s, Pack Of 20<br>Jps Black 100s, Pack Of 20
Jps Stream Blanc	Jps White, Pack Of 20<br>Jps Classic Stream White, Pack Of 20
Jps Firm Filter Noir	Jps Classic Black, Pack Of 20<br>Jps Black, Pack Of 20
Jps Stream Bleu	Jps Classic Blue, Pack Of 20
Jps Firm Filter Rouge 100's	Jps Classic Red 100s, Pack Of 20<br>Jps Red 100s, Pack Of 20
Jps Firm Filter Rouge	Jps Classic Firm Filter Red, 20 Pack<br>Jps Classic Red, Pack Of 20
L&M Blue Xxl Hard Pack - 30 Cigarettes	L&M Blue Xxl, Pack Of 30<br>L&M International Blue Xxl, Pack Of 30
L&M Blue Xl Hard Pack - 25 Cigarettes	L&M Blue Xl, Pack Of 25
L&M Blue Hard Pack - 20 Cigarettes	L&M Blue, Pack Of 20
L&M Red 100s 100s Hard Pack - 20 Cigarettes	L&M International Red 100s, Pack Of 20<br>L&M Red 100s, Pack Of 20
L&M Red Xxl Hard Pack - 30 Cigarettes	L&M International Red Xxl, Pack Of 30
L&M Red Hard Pack - 20 Cigarettes	L&M International Red, Pack Of 20<br>L&M Red, Pack Of 20
L&M Red Xl Hard Pack - 25 Cigarettes	L&M Red Xl, Pack Of 25
Lucky Strike Bleu	Lucky Strike Blue Blend, Pack Of 20<br>Lucky Strike Classic Blue, Pack Of 20<br>Lucky Strike Blue Select, Pack Of 20<br>Lucky Strike Blue, Pack Of 20<br>Lucky Strike Ice Light Blue Clear Ice Pack Of 20<br>Lucky Strike Ice Light Blue, Pack Of 20<br>Lucky Strike Ice Blue, Pack Of 20
Lucky Strike Gold	Lucky Strike Gold Blend, Pack Of 20<br>Lucky Strike Gold, Pack Of 20
Lucky Strike Alaska	Alaska Select Lucky Strike Ice, Pack Of 20<br>Lucky Strike Ice Alaska, 20 Pack<br>Lucky Strike Ice Alaska Ice Cream, 20 Pack

Lucky Strike Double	Lucky Strike Double Ice Ice Pack Of 20
Lucky Strike Verte	Lucky Strike Ice Frozen Green, Pack Of 20<br>Lucky Strike Ice Green Select, Pack Of 20<br>Lucky Strike Ice Green, Pack Of 20
Lucky Strike Red 100's	Lucky Strike Red Long (100s), Pack Of 20<br>Lucky Strike Red Select Long, 20 Pack<br>Lucky Strike Red Long Blend, Pack Of 20
Lucky Strike Original Red	Lucky Strike Original Red American Blend In 20<br>Lucky Strike Original, Pack Of 30
Lucky Strike Original Tobacco Red	Lucky Strike Original Red 100% Select Tobacco In 20
Lucky Strike Red	Lucky Strike Red Blend, Pack Of 20
Lucky Strike Red	Lucky Strike Red Blend, Pack Of 40<br>Lucky Strike Red, Pack Of 40
Marlboro (Red) Duty Free - Hard Pack - 20 Cigarettes	Marlboro Red<br>Marlboro<br>Marlboro Red (Rigid Package), Pack Of 20
Marlboro Gold Duty Free - Hard Pack - 20 Cigarettes	Marlboro Gold<br>Marlboro Gold (Rigid Package), 20 Pack
Marlboro Gold 100s 100s Hard Pack - 20 Cigarettes	Marlboro Gold 100s (Rigid Package), Pack Of 20
Marlboro Gold Slims 100s Hard Pack - 20 Cigarettes	Marlboro Gold Slims, Pack Of 20
Marlboro Red 100s Hard Pack - 20 Cigarettes	Marlboro Red 100s (Rigid Package), Pack Of 20
Marlboro Gold Xl Hard Pack - 25 Cigarettes	Marlboro Xl Classic Gold, Pack Of 25
Marlboro Red Xl Hard Pack - 25 Cigarettes	Marlboro Xl Classic Red, Pack Of 25
Marlboro Xxl Classic Gold Hard Pack - 30 Cigarettes	Marlboro Xxl Classic Gold, Pack Of 30
Marlboro Xxl Classic Red Hard Pack - 30 Cigarettes	Marlboro Xxl Classic Red, Pack Of 30
News & Co Rouge	News & Co Classic Red, 20 Pack<br>News & Co Classic Red, Pack Of 40<br>News & Co Red, Pack Of 20<br>News & Co Red, Pack Of 40
News Rouge	News Rouge<br>Red News, Pack Of 20<br>News Red, Pack Of 25<br>Red News, Pack Of 30<br>News Rouge X30
News Marron	Brown News, Pack Of 20
Pall Mall Rouge	Pall Mall Rouge Blend, Pack Of 20<br>Pall Mall Red, Pack Of 20
Peter Stuyvesant Red 100's	Peter Stuyvesant Red Long (100s), Pack Of 20
Peter Stuyvesant Rouge	Peter Stuyvesant Red<br>Peter Stuyvesant Red Select, Pack Of 20<br>Peter Stuyvesant Red, Pack Of 20

Peter Stuyvesant Silver	Peter Stuyvesant Silver Select, Pack Of 20<br>Peter Stuyvesant Silver, Pack Of 20
Peter Stuyvesant Blue 100's	Peter Stuyvesant Blue Long (100s), Pack Of 20
Peter Stuyvesant Blue	Peter Stuyvesant Blue, Pack Of 20
Peter Stuyvesant Verte	Peter Stuyvesant Green, Pack Of 20
Philip Morris Bleue Duty Free - Hard Pack - 20 Cigarettes	Philip Morris Blue<br>Philip Morris Blue, Pack Of 20
Philip Morris Bleue 100s 100s Hard Pack - 20 Cigarettes	Philip Morris Blue 100s, Pack Of 20
Philip Morris Filter Kings Duty Free - Hard Pack - 20 Cigarettes	Philip Morris Filter Kings (Rigid Package) In 20
Philip Morris Filter Kings Soft Pack - 20 Cigarettes	Philip Morris Filter Kings (Soft Pack) In 20
Philip Morris Filter Kings 100s Hard Pack - 20 Cigarettes	Philip Morris Filter Kings 100s, Pack Of 20
Philip Morris Green Hard Pack - 20 Cigarettes	Philip Morris Green, Pack Of 20
Philip Morris White Silver Hard Pack - 20 Cigarettes	Philip Morris White Silver, Pack Of 20
Philip Morris Xl Hard Pack - 25 Cigarettes	Philip Morris Xl, Pack Of 25
Philip Morris Xxl Hard Pack - 30 Cigarettes	Philip Morris Xxl, Pack Of 30
Rothmans Red	Rothmans Rouge Blend, Pack Of 20<br>Rothmans Red Select, Pack Of 20<br>Rothmans Red, Pack Of 20
Rothmans Blue	Rothmans Blue Blend, Pack Of 20<br>Rothmans Blue, Pack Of 20
Rothmans Blue 100's	Long Blue Rothmans, 20 Pack
Royale By Davidoff Blanc	Royale By Davidoff Menthol White, Pack Of 20<br>Royale By Davidoff White, Pack Of 20
Virginia Slims 100s Hard Pack - 20 Cigarettes	Virginia Slims No 602, Pack Of 20
Vogue Bleue	Vogue Lessentiel Bleue Select, Pack Of 20<br>Vogue Lessentiel Blue, Pack Of 20
Vogue Pastel	Vogue Pastel Essential, Pack Of 20<br>Vogue Loriginale Pastel, Pack Of 20<br>Vogue Loriginale Pastel Select, Pack Of 20
Winfield Rouge	Winfield Rouge X 30<br>Winfield Red Basic, Pack Of 30<br>Winfield Rouge Classic, Pack Of 30
Winfield Rouge	Winfield Red Basic, Pack Of 20
Winfield Blue 30	Winfield Basic Blue, Pack Of 30<br>Winfield Blue, Pack Of 30
Winston Classic	Winston Classic (Rigid Package), Pack Of 20<br>Winston Classic (Soft Pack), 20 Pack
Winston Classic 100's	Winston Classic 100s, Pack Of 20<br>Winston Classic 100s, Pack Of 30
Winston Red	Winston Original Red, Pack Of 25<br>Winston Original Red, Pack Of 30

Winston White	Winston Original White, Pack Of 20<br>Winston White, Pack Of 20
Winston Xsphere Fresh 100's	Winston Xsphere 100s, Pack Of 20
Winston Ssl	Winston Original Ssl, Pack Of 20<br>Winston Ssl, Pack Of 20

Supplemental Table S2. Correlations between cigarette design features, 2019 ITC France Survey

		Pressure Drop Open (mmWG)	Pressure Drop Closed (mmWG)	Ventilation (0-100%)	Tar (mg)	Nicotine (mg)	CO (mg)	Product Length (mm)	Diameter (mm)	Wet Tobacco Weight (mg)	Dry Tobacco Weight (mg)	Filter Length (mm)
**Pressure Drop Open (mmWG)**	**r**	1	0.27	-0.17	-0.15	-0.4	0.18	0.26	-0.08	-0.11	-0.33	0.05
	**p**		<0.001	<0.001	<0.001	<0.001	<0.001	<0.001	0.01	<0.001	<0.001	0.06
**Pressure Drop Closed (mmWG)**	**r**	0.27	1	0.65	-0.31	-0.02	-0.18	0.48	-0.68	0.4	0.11	0.43
	**p**	<0.001		<0.001	<0.001	0.29	<0.001	<0.001	<0.001	<0.001	<0.001	<0.001
**Ventilation (0-100%)**	**r**	-0.17	0.65	1	-0.42	-0.18	-0.28	0.51	-0.63	0.56	0.19	0.47
	**p**	<0.001	<0.001		<0.001	<0.001	<0.001	<0.001	<0.001	<0.001	<0.001	<0.001
**Tar (mg)**	**r**	-0.15	-0.31	-0.42	1	0.73	0.73	-0.05	0.18	-0.03	0.29	-0.71
	**p**	<0.001	<0.001	<0.001		<0.001	<0.001	0.05	<0.001	0.22	<0.001	<0.001
**Nicotine (mg)**	**r**	-0.4	-0.02	-0.18	0.73	1	0.28	-0.05	-0.04	0.02	0.46	-0.44
	**p**	<0.001	0.29	<0.001	<0.001		<0.001	0.05	0.09	0.3	<0.001	<0.001
**CO (mg)**	**r**	0.18	-0.18	-0.28	0.73	0.28	1	0.22	0.16	0.15	0.01	-0.65
	**p**	<0.001	<0.001	<0.001	<0.001	<0.001		<0.001	<0.001	<0.001	0.43	<0.001
**Product Length (mm)**	**r**	0.26	0.48	0.51	-0.05	-0.05	0.22	1	-0.44	0.57	0.21	0.37
	**p**	<0.001	<0.001	<0.001	0.05	0.05	<0.001		<0.001	<0.001	<0.001	<0.001
**Diameter (mm)**	**r**	-0.08	-0.68	-0.63	0.18	-0.04	0.16	-0.44	1	-0.36	-0.12	-0.3
	**p**	0.01	<0.001	<0.001	<0.001	0.09	<0.001	<0.001		<0.001	<0.001	<0.001
**Wet Tobacco Weight (mg)**	**r**	-0.11	0.4	0.56	-0.03	0.02	0.15	0.57	-0.36	1	0.56	0.36
	**p**	<0.001	<0.001	<0.001	0.22	0.3	<0.001	<0.001	<0.001		<0.001	<0.001
**Dry Tobacco Weight (mg)**	**r**	-0.33	0.11	0.19	0.29	0.46	0.01	0.21	-0.12	0.56	1	0.03
	**p**	<0.001	<0.001	<0.001	<0.001	<0.001	0.43	<0.001	<0.001	<0.001		0.17
**Filter Length (mm)**	**r**	0.05	0.43	0.47	-0.71	-0.44	-0.65	0.37	-0.3	0.36	0.03	1
	**p**	0.06	<0.001	<0.001	<0.001	<0.001	<0.001	<0.001	<0.001	<0.001	0.17	

r = correlation coefficient. p = p-value.

Supplemental Table S3A. Cigarette design features (pressure drop open, pressure drop closed, ventilation, and product length) by participant characteristics and outcome measures, 2019 ITC France Survey

	Pressure Drop Open (mmWG)			Pressure Drop Closed (mmWG)			Ventilation (0-100%)			Product Length (mm)		
	Mean	F (n, d)	p	Mean	F (n, d)	p	Mean	F (n, d)	p	Mean	F (n, d)	p
**Age (years)**												
18-34	82.97	5.15 (3, 969)	0.002	87.32	12.47 (3, 969)	<0.001	28.00	7.62 (3, 969)	<0.001	83.07	8.94 (3, 969)	<0.001
35-44	87.91			92.15			29.78			84.16		
45-54	86.18			96.59			31.96			85.73		
≥55	86.49			107.18			33.02			85.27		
**Gender**												
Male	84.86	1.07 (1, 971)	0.30	87.56	31.08 (1, 971)	<0.001	28.50	15.78 (1, 971)	<0.001	83.71	7.30 (1, 971)	0.01
Female	85.92			100.68			31.85			84.86		
**Cigarettes per day**												
0-10	85.91	2.96 (2, 962)	0.05	94.74	0.65 (2, 962)	0.52	30.55	1.56 (2, 962)	0.21	84.21	1.42 (2, 962)	0.24
11-20	85.47			93.89			30.03			84.57		
21+	81.05			89.42			27.64			83.15		
**Experience with own brand vs. other brands**												
Harsher/Same	84.54	3.10 (1, 848)	0.08	92.27	4.16 (1, 848)	0.04	28.97	11.34 (1, 848)	0.001	83.63	11.00 (1, 848)	0.001
Smoother	86.62			97.84			32.21			85.21		
**Perception of how harmful own brand is vs. other brands**												
No different or a little more	85.21	8.79 (1, 852)	0.003	93.03	2.44 (1, 852)	0.12	30.05	0.90 (1, 852)	0.34	84.23	0.70 (1, 852)	0.40
A little less	90.77			99.93			31.51			84.87		

F (n, d) = F-statistic (numerator degrees of freedom, denominator degrees of freedom). p = p-value.

Supplemental Table S3B. Cigarette design features (diameter, wet tobacco weight, dry tobacco weight) by participant characteristics and outcome measures, 2019 ITC France Survey

	Diameter (mm)			Wet Tobacco Weight (mg)			Dry Tobacco Weight (mg)		
	**Mean**	**F (n, d)**	**p**	**Mean**	**F (n, d)**	**p**	**Mean**	**F (n, d)**	**p**
**Age (years)**									
18-34	7.76	3.15 (3, 969)	0.02	832.07	5.72 (3, 969)	0.001	620.34	2.65 (3, 957)	0.05
35-44	7.73			835.25			614.57		
45-54	7.77			857.38			630.44		
≥55	7.68			848.95			629.27		
**Gender**									
Male	7.78	11.13 (1, 971)	0.001	839.49	0.48 (1, 971)	0.49	625.17	1.23 (1, 959)	0.27
Female	7.70			842.97			620.37		
**Cigarettes per day**									
0-10	7.71	4.97 (2, 962)	0.01	833.12	6.81 (2, 962)	0.001	617.01	4.92 (2, 951)	0.01
11-20	7.78			852.08			629.01		
21+	7.80			848.65			636.07		
**Experience with own brand vs. other brands**									
Harsher/Same	7.74	0.22 (1, 848)	0.64	835.88	3.54 (1, 848)	0.06	622.68	0.08 (1, 837)	0.78
Smoother	7.73			846.46			621.32		
**Perception of how harmful own brand is vs. other brands**									
No different or a little more	7.75	8.09 (1, 852)	0.01	841.95	4.24 (1, 852)	0.04	623.51	5.69 (1, 842)	0.02
A little less	7.63			823.00			604.38		

F (n, d) = F-statistic (numerator degrees of freedom, denominator degrees of freedom). p = p-value.

Supplemental Table S3C. Cigarette design features (tar, nicotine, CO) by participant characteristics and outcome measures, 2019 ITC France Survey

	Tar (mg)			Nicotine (mg)			CO (mg)		
	**Mean**	**F (n, d)**	**p**	**Mean**	**F (n, d)**	**p**	**Mean**	**F (n, d)**	**p**
**Age (years)**									
18-34	9.39			0.74			9.20		
35-44	9.21	10.53 (3, 969)	<0.001	0.72	5.73 (3, 969)	0.001	9.21	6.78 (3, 969)	<0.001
45-54	9.03			0.71			9.14		
≥55	8.61			0.70			8.64		
**Gender**									
Male	9.22	3.03 (1, 971)	0.08	0.72	0.04 (1, 971)	0.84	9.12	0.52 (1, 971)	0.47
Female	9.04			0.72			9.05		
**Cigarettes per day**									
0-10	9.04			0.72			8.99		
11-20	9.22	2.02 (2, 962)	0.13	0.73	1.23 (2, 962)	0.29	9.21	2.36 (2, 962)	0.09
21+	9.34			0.74			9.12		
**Perceived health status**									
Poor or fair	9.24	1.89 (1, 940)	0.17	0.73	1.90 (1, 940)	0.17	9.24	5.05 (1, 940)	0.02
Other (good, very good, or excellent)	9.08			0.72			9.01		
**Experience with own brand vs. other brands**									
Harsher/Same	9.27	13.44 (1, 848)	<0.001	0.74	15.22 (1, 848)	<0.001	9.15	4.45 (1, 848)	0.04
Smoother	8.84			0.70			8.92		
**Perception of how harmful own brand is vs. other brands**									
No different or a little more	9.20	11.44 (1, 852)	0.001	0.73	12.95 (1, 852)	<0.001	9.15	6.83 (1, 852)	0.01
A little less	8.57			0.67			8.69		

F (n, d) = F-statistic (numerator degrees of freedom, denominator degrees of freedom). p = p-value.

Supplemental Table S4. Logistic regression of factors associated with intention to quit, 2019 ITC France Survey (n=835)

Variables	OR [95% CI]	Wald Chi-Square	p-value
**Ventilation (0-100%)**	1.00 [0.99, 1.02]	0.21	0.64
**Age (years)**			
18-34	1.92 [1.23, 3.00]	8.14	0.004
35-44	1.51 [0.92, 2.48]	2.71	0.10
45-54	1.30 [0.80, 2.12]	1.15	0.28
≥55	1 (referent)		
**Gender**			
Male	1.05 [0.75, 1.46]	0.07	0.79
Female	1 (referent)		
**Highest level of education**			
Low (no education, some high school)	1.07 [0.66, 1.73]	0.08	0.78
Moderate (completed high school, some university)	1.07 [0.65, 1.75]	0.07	0.79
High (completed university, Ph.D. and/or post-doc)	1 (referent)		
**Cigarettes per day**			
0-10	1 (referent)		
11-20	1.02 [0.72, 1.44]	0.01	0.92
21+	0.99 [0.51, 1.92]	0.00	0.97
**Perceived health status**			
Poor or fair	1.21 [0.84, 1.74]	1.06	0.30
Other (good, very good, or excellent)	1 (referent)		

OR [95% CI] = odds ratio [95% confidence interval]. Model adjusted for age, gender, highest level of education, cigarettes per day, perceived health status, and ventilation.

© 2025 Polston P.A. et al.
